# Association of MOS-Based Blast Exposure With Medical Outcomes

**DOI:** 10.3389/fneur.2020.00619

**Published:** 2020-07-31

**Authors:** Walter Carr, Amanda L. Kelley, Christine F. Toolin, Natalya S. Weber

**Affiliations:** ^1^Center for Military Psychiatry and Neuroscience, Walter Reed Army Institute of Research, Silver Spring, MD, United States; ^2^Oak Ridge Institute for Science and Education, Oak Ridge, TN, United States

**Keywords:** blast, military, epidemiology, occupation, healthcare

## Abstract

The study of effects associated with human exposure to repeated low-level blast during training or operations of select military occupational specialties (MOS) challenges medical science because acute negative effects that might follow such exposures cannot be expected to be clear or prevalent. Any gross effects from such occupational blast exposure on health or performance should be expected to have been already identified and addressed by affected military units through changes to their standard training protocols. Instead, effects, if any, should be expected to be incremental in nature and to vary among individuals of different susceptibilities and exposure histories. Despite the challenge, occupational blast-associated effects in humans are emerging in ongoing research. The purpose of the present study was to examine medical records for evidence of blast-associated effects that may have clinical significance in current standard of care. We hypothesized that populations exposed to blast by virtue of their military occupation would have poorer global medical outcomes than cohorts less likely to have been occupationally exposed. Records from a population of 50,254 service members in MOSs with a high likelihood of occupational blast exposure were compared to records from a matched cohort of 50,254 service members in MOSs with a lower likelihood of occupational blast exposure. These two groups were compared in hospitalizations, outpatient visits, pharmacy, and disability ratings. The clearest finding was higher risk among blast-exposed MOSs for ambulatory encounters for tinnitus, with adjusted risk ratios of 1.19 (CI 1.03–1.37), 1.21 (CI 1.16–1.26), and 1.31 (CI 1.18–1.45) across career time points. Other hypothesized effects (i.e., neurological outcomes) were smaller and were associated with acute exposure. This study documents that service members in occupations that likely include repeated exposure to blast are at some increased risk for neurosensory conditions that present in medical evaluations. Other hypothesized risks from occupational exposure may manifest as symptomology not visible in the medical system or current standard of care. Separate studies, observational and epidemiological, are underway to evaluate further the potential for occupational risk, but the evidence presented here may indicate near-term opportunities to guide efforts to reduce neurosensory risk among exposed service members.

## Introduction

The term “occupational blast exposure” is intended to denote repeated exposures to low-level explosive blast events that occur as part of training and operational activities experienced by personnel in designated roles in the military and law enforcement. Such roles include indirect fires (artillery, mortar), explosive breaching, and antiarmor weapon operation. These occupational roles will differ in blast exposure magnitude, frequency, periodicity, or concomitant factors such as acoustic insult, aerobic exertion, and psychological stress. The common factor across occupational roles, *repeated blast exposure*, has been of increasing concern as a cause of negative neurological effects, especially in context of similar increasing concern for brain injury from repeated head impacts in contact sports like American football. Exposure to occupational blast (i.e., low-level blast) is not known to result in acute injury; neurological effects, if any, would be cumulative in nature and not recognizable as diagnosis of traumatic brain injury (TBI) in standard of care medicine. Anecdotal reports suggest that service members with particularly high levels of occupational blast exposure, chronic exposure, experience negative neurological effects, but relevant research has not shown conclusive evidence of such effects (1, 2). The work presented here is an examination of medical records for clinical evidence for hypothesized occupational blast exposure effects.

After years of repeated occupational exposure to explosive events used in close proximity (high explosive or propellant combustion in heavy weapons), some individuals report symptoms consistent with concussion (e.g., memory deficits, headache, dizziness, difficulty concentrating). Those symptoms are reported as experienced to a greater degree during periods of repeated exposure to blast in training. The anecdotally reported occupational blast-related symptomology has been supported by a symptom survey among a blast-exposed professional community (3), by pilot study evidence that included cognitive performance and blood-based neurotrauma biomarkers collected during training programs involving explosives (4), and by symptom inventory in other field studies of operational training (5–9). In addition to symptom reporting, research observations of low-level blast-associated effects have included deficits in cognitive function (10), cellular changes in peripheral blood (11–15), and neuroimaging evidence that blast exposure may negatively affect neurophysiological functioning in simple tasks requiring memory of visual stimuli (6). It is important to note that none of these cited studies included blast exposure association with diagnosed injury—the focus was on blast exposures considered low level in magnitude. This growing body of evidence is suggestive of an association between occupations that have a likelihood of repeated exposure to explosive blast and negative effects on health, but the entirety of this evidence has been subclinical, unassociated with medical diagnosis of injury.

In contrast, populations that do receive clinical diagnosis of TBI following acute exposure to significant blast events in a combat setting show clear evidence of blast-related neurotrauma, directly supporting the diagnosis. The clinical relevance of repeated low-level blast, such as experienced in routine training in some occupational specialties, is unknown. Effects from such low-level exposures may present differently than effects from acute exposure to significant blast events and thus, are not identified as diagnosable TBI but may be diagnosed as other conditions. A corollary condition may be chronic traumatic encephalopathy, recently observed among athletes in contact sports who sustain many hundreds or thousands of subconcussive blunt impacts to the head (16). Chronic traumatic encephalopathy, or other neuropathology from repeated subconcussive events, can present as symptomology during life but is not diagnosed until after death, upon postmortem exam. Research is needed to understand the clinical presentation of such conditions in standard of care medicine. Longitudinal or cross-sectional comparisons among cohorts of interest would be an important addition to current findings. Here, we addressed that gap for the risk associated with repeated exposure to low-level blast, which is not known to be currently associated with a clinical diagnosis. Professional communities exposed to blast in their occupational roles may have exposures during tactical operations, but they will all have exposures during routine training, for acquiring needed skills as well as maintaining those skills over time. Occupation-based estimates of risk from exposure history have been revealed for military occupational specialties (MOSs) in previous studies (17–19) and could serve to prevent injury as has been recommended for contact sports (20).

This study was a subproject to the Accession Medical Standards Analysis and Research Activity (AMSARA) CORE protocol (21) and utilized data already collected for other purposes from AMSARA and the Tri-Service Disability Evaluation Systems Database Analysis and Research (DES), which is part of the same contract as AMSARA.

## Materials and Methods

### Study Design

This matched cohort study compared healthcare utilization, prescription drug utilization, and disability discharge between Soldiers with specific combat arms occupations, with MOS serving as a proxy for occupational exposure to explosive blast, vs. Soldiers with occupations that are likely to deploy to a combat zone but less likely to be occupationally exposed to blast. The inclusion of several categories of medical outcomes in this design was to increase sensitivity of finding an occupation-based chronic exposure effect not associated with acute diagnosis, within datasets that are coded according to diagnoses. To assess the short-, medium-, and long-term effects of occupational exposure to blast, three time periods of military service were used for ascertaining the study outcomes: first 12 months, from 1 to 7 years, and from 8 to 14 years of service. These time periods will reflect conditions at baseline and initial training, conditions from a full tour of duty, and conditions beyond one tour of duty.

This study was performed under a minimal risk human use WRAIR protocol (#2023.05) reviewed and approved by the Walter Reed Army Institute of Research Institutional Review Board.

### Study Population

All active duty enlisted US Army men who initially entered service from fiscal year (FY) 2000 to 2013 (October 1, 1999 to September 30, 2013) were eligible for inclusion in this study. Records prior to 1999 were not included because there was less consistent digitization of records at those earlier dates. Records later than 2013 were not included because this study was initially designed in 2015, and AMSARA uses a 2-year time lag in epidemiological studies to accommodate time for medical records to be completed, digitized, and centralized.

Eligible Soldiers for the Exposed group were excluded if they received a preaccession disqualification or medical waiver for tinnitus, headache, or sleep disturbance, or if their records were missing any variables that were of interest in this study. Those hospitalized for severe or penetrating TBI or traumatic amputation were excluded because these injuries indicated a single exposure to a high-energy blast. The primary purpose of this study was to examine the health effects of Soldiers occupationally exposed to blast over time and without clearly associated diagnosis from acute blast exposure—to include Soldiers with major medical conditions or diagnosed injuries directly associated with high-energy blast events would bias the results and would not be consistent with the primary purpose of the study. TBI that was not severe was not a criterion for exclusion. Excluding Soldiers with mild TBI from the study would have made the population less representative of the MOSs. The population of Soldiers eligible for the Exposed group had military occupations that were likely to be occupationally exposed to blast by virtue of MOS descriptions and training required for major duties and included Cannon Crewmembers, Explosive Ordinance Disposal Specialists, Indirect Fire Infantrymen, Combat Engineers, and Special Forces. Descriptions for major duties of these MOSs are available in the Department of the Army Pamphlet 611–21 (Smartbook) (22) and are reflected in a number of other sources, including public domain websites (e.g., army-portal.com). Each of the five MOSs listed here has descriptions that stipulate explosives or heavy weapons in the basic level of MOS major duties (see Table 1 for example).

**Table 1:** Department of the Army Pamphlet 611–21 (Smartbook) (2017) “Military Occupational Classification and Structure” descriptions of basic level major duties for example MOS in the Exposed and the Unexposed groups.

Exposed group example MOS **[emphasis added]:** 10–13B. MOS 13B—Cannon Crewmember, CMF 13 a. Major duties. The cannon crewmember supervises or serves as **a member of field artillery cannon section** or ammunition section. (1) MOSC 13B1O. Integral member of a crew that operates high technology cannon artillery weapon systems. Load and **fire howitzers**. Sets fuse and charge on a variety of munitions, including high explosive artillery rounds, laser guided projectiles, scatterable mines, and rocket assisted projectiles. Uses computer generated fire direction data to set elevation of cannon tube for loading and firing. **Employ rifles, machine guns, and grenade and rocket launchers in offensive and defensive operations**. Drives and operates heavy and light wheeled trucks and tracked vehicles. Transports and manages artillery ammunition. Participate in reconnaissance operations to include security operations and position preparation. Operate in reduced visibility environments with infrared and starlight enhancing night vision devices and other equipment. Coordinate movement into position. Camouflages position area. Communicate using voice and digital wire and radio equipment. Use critical combat survival skills to operate in a hostile environment. Maintain operational readiness of vehicles and equipment.
Unexposed group example MOS: 10–92A. MOS 92A—Automated Logistical Specialist (Auto Log Spec) CMF 92 a. Major duties. The automated logistical specialist supervises and performs management or stock record/warehouse functions pertaining to receipt, storage, distribution, and issue and maintains equipment records and parts. Duties for MOS 92A at each level of skill are: (2) MOSC 92A1O. Establishes and maintains stock records and other documents such as inventory, materiel control, accounting and supply reports. Establishes and maintains automated and manual accounting records, posts receipts, and turn-ins and performs dues-ins and dues-outs accounting. Correct error and exception documents. Reviews and verifies quantities received against bills of lading, contracts, purchase requests and shipping documents. Unloads, unpacks, visually inspects, counts, segregates, palletizes, and stores incoming supplies and equipment. Maintains stock locator system and administers document control procedures. Repairs and constructs fiberboard or wooden containers. Packs, crate, stencil, weigh and band equipment and supplies. Construct bins, shelving and other storage aids. Processes request, and turn-in documents at direct support level through warehousing section. Processes inventories, surveys and warehousing documents. Performs prescribed load list (PLL) and shop stock list (SSL) duties in manual and automated supply applications. Prepares, annotates and distributes shipping documents. Breaks down and distributes field rations. Operate material handling equipment (MHE). Perform accounting and sales functions in self-service supply. Perform Standard Army Maintenance System Enhanced (SAMS-E) duties in automated applications. Simplifies and standardizes the collection and use of maintenance data. Improves readiness management and visibility by providing equipment status and asset data. Raise the quality and accuracy of performance, cost, backlog, man-hour, and parts data through improved maintenance management.

Soldiers eligible for the Unexposed group had military occupations that were likely to deploy to a combat zone but less likely to be occupationally exposed to blast, especially during training. These occupations included Quartermaster, Military Intelligence, Signal, Field Mechanical Maintenance, Engineers other than combat, Psychological Operations, or those who are Motor Transport Operators, Radar Operators, Military Police, or Chemical/Biological/Radiological/Nuclear Specialists. Eligible Soldiers for the Unexposed group were individually matched to the Exposed group on fiscal year of and age at military entry and history of deployment (yes/no). Those matched Soldiers were then randomly sampled to yield an equivalent number for the Unexposed group.

In the three stratified time periods (first 12 months, years 1–7, years 8–14) for both groups, the first time period included the full study population, while the second time period included only Soldiers who did not attrit within the 1st year of service. The third time period (years 8–14) includes those who entered military service prior to FY 2008 and have at least 8 years of military service.

### Data Sources

The Defense Manpower Data Center (DMDC), Seaside, CA, provided entry dates, loss dates, deployment dates, and locations, military occupation, age, sex, race, education, and marital status. The US Military Entrance Processing Command (USMEPCOM) provided data from study subjects' medical examination prior to military entry, specifically examination dates, medical qualification status (fully qualified, medical disqualification, administrative qualification), and where relevant, medical diagnoses based on International Classification of Diseases, Ninth Revision (ICD-9) codes. The US Army Recruiting Command, Fort Knox, Kentucky provided data on recruits who had a medical disqualification at the pre-enlistment medical examination and sought a medical waiver. These data included medical waiver action (approved, denied) and disease/disorder in the form of ICD-9 diagnosis codes.

Data on medical encounters occurring at military treatment facilities (MTFs) during the study period were provided by the Defense Health Agency, and prescriptions filled at MTFs since 2002 were provided by the Pharmacy Data Transaction Service via the Military Health System (MHS) Data Repository. These data included encounter dates, count of bed days, and ICD-9 disease/disorder codes for each medical encounter or fill dates, drug type, drug category, and days supply for pharmacy data.

Data on disability discharge considerations were provided by the US Army Physical Disability Agency (PDA) and included demographic characteristics at the time of disability evaluation as well as information pertaining to the disability evaluation including dates, disposition, percent rating, and the diseases or disorders for which the Soldier was deemed unfit. Diseases and disorders present at disability evaluations are coded based on the Veterans Affairs Schedule for Rating Disabilities (VASRD) in lieu of ICD-9 codes.

### Measures

The three time periods of military service (first 12 months, from 1 to 7 years, and from 8 to 14 years of service) were calculated using the date of the Soldier's first military entry and date of the study outcome, which included the date of the healthcare encounter, the date of the prescription fill, or the date of disability discharge. Time in years to first deployment was calculated as the duration between the date the Soldier's initial military entry and date of his first deployment. Time in months on deployment was calculated as duration of every unique deployment, using deployment begin and end dates. Length of service was the duration between a Soldier's earliest date of military entry and the most recent date of military exit. A Soldier was categorized as having been deployed if deployed in support of Overseas Contingency Operations/Global War on Terrorism at any time during the study period. Deployment count was determined by the number of unique deployments based on deployment date.

Overall healthcare utilizations, including the total number with an encounter and the average number of encounters per person, were determined by counting all unique hospitalizations or ambulatory encounters for Soldiers with at least one encounter for each time period. Ambulatory encounters were identified as unique using both date and appointment identification number, allowing for multiple ambulatory encounters on the same day. Inpatient encounters were counted as unique by date of admission; however, an admission date within a week of a prior hospitalization was counted as one hospitalization. Previous examination of data from military hospitals found hospital readmissions within 7 days of a prior admission's discharge date were most likely to be a transfer to another military hospital.

Specific disorders, disease or disorder subcategories by system, and drug classes were chosen as outcomes of interest based on the etiopathophysiological pathways consistent with or possibly related to the consequences of blast exposure. The proportion of Soldiers with a healthcare encounter and the average number of encounters per specific disorder or subcategory were calculated using all unique hospitalizations or ambulatory encounters for Soldiers with at least one encounter for that specific disorder or subcategory in any diagnostic position stratified by time period. All bed days for all hospitalizations per Soldiers were totaled to examine the average number of bed days overall and by specific disorder or subcategory for each time period. Due to the large number of prescription drug classes, drug classes were combined into general therapeutic classes (e.g., cardiac drugs). The general therapeutic classes examined in this study were skeletal muscle relaxants, cardiac drugs, hypotensive agents, vasodilating agents, central nervous system agents, analgesics/antipyretics, anticonvulsants, psychotherapeutic agents, and anxiolytics/sedatives/hypnotics. The total number of fills and the total number of days supply for prescription drugs, both overall and by therapeutic class, were calculated by counting all unique drug fills, based on the date of the drug fill and the name of the drug, for each time period.

A Soldier may be considered unfit for military service and disability discharged due to either a single disease/disorder or their combined effect. The medical conditions are based on the VASRD codes, which were designed for the purpose of disability rating and compensation rather than medical diagnoses, and do not necessarily correlate directly to ICD-9 diagnostic codes. In this study, multiple VASRDs were combined to create the disability subcategories of interest, which were based on diseases or disorders possibly related to the consequences of blast exposure. A total disability rating, calculated by the PDA, is based on disability ratings for each individual medical condition and is expressed as a percentage. The total disability rating is then used to assign a disability disposition. Service members receiving a rating of 20% or less are usually separated with a one-time severance payment, while those receiving a rating of 30% or greater are eligible for disability retirement benefits, which include lifetime monthly retirement pay and access to MHS medical care. The disability evaluation board may deem a Soldier's medical condition(s) as not unfitting; these Soldiers are designated as fit and may continue military service. Soldiers may be evaluated for disability more than once, particularly if the severity of their disease or disorder could change over time. In these cases, the final disposition, disability rating, and medical conditions were collected from the most recent disability record.

### Statistical Analysis

Univariate analysis was used to characterize and compare the distribution of variables of interest between the exposed and unexposed populations. Frequencies and proportions were utilized to examine the categorical variables, including demographic (e.g., race), military (e.g., MOS), deployment (e.g., number of deployments), and disability discharge (e.g., disability rating) characteristics. Means and standard deviations were calculated for continuous variables, including total length in service, total deployment duration, total number of conditions at disability evaluation, and length in service to first deployment or to disability evaluation. Cox proportional hazards regression models calculated crude and adjusted relative risks and associated 95% confidence intervals and were utilized to determine which factors are more likely to occur in the Exposed group than the Unexposed group (23). The adjusted regression models controlled for potential confounding from race category or educational level at military entry.

Univariate analysis was also used to compare healthcare utilization patterns, including the average number of healthcare encounters and the average number of bed days, as well as prescription drug utilization, including the average number of fills and the average days' supply, which were characterized overall and by group of pathology (e.g., respiratory) or specific nosology (e.g., headaches). To assess whether certain disorders, disease or disorder subcategories by system, or prescription medication use are significantly more common among those occupationally exposed to blast from a clinical standpoint, relative risks, and associated 95% confidence intervals were calculated using Cox proportional hazards regression. Adjusted relative risks and associated 95% confidence intervals, controlling for race and education, were also calculated.

All statistical analyses were performed using SAS version 9.4 (SAS Institute, Cary, NC). Given that results are reported as risk ratios and confidence intervals, *p*-values are not reported separately.

## Results

Table 2 describes and compares the demographic, deployment, and military characteristics of the population occupationally exposed to blast (Exposed group; *n* = 50,254) and the population less likely to be occupationally exposed to blast (Unexposed group; *n* = 50,254). The occupation with the largest proportion within the Exposed group was Cannon Crewmember (43%), followed by Combat Engineer and Special Forces, which each comprised 20% of the Exposed group. The most common occupations in the Unexposed group were Quartermaster (29%), Field Mechanical Maintenance (21%), Signal (14%), Motor Transport Operators (11%), and Military Intelligence (11%). The group occupationally exposed to blast was slightly more likely to be white and have a higher education level at military entry than the Unexposed group. Of those who were deployed, both groups were deployed a similar number of times, yet the Exposed group was deployed, on average, slightly earlier (Exposed = 1.75 years of service; Unexposed = 1.90 years of service) and for a slightly longer duration (Exposed = 15.2 months; Unexposed = 14.8 months) than the Unexposed group. Soldiers occupationally exposed to blast had a slightly shorter time in military service than those unlikely to be exposed to blast (Exposed = 4.5 years; Unexposed = 4.9 years). In regard to attrition for each group across the three time periods, attrition rates for Exposed and Unexposed groups at years 1–7 were 11 and 9%, respectively, and at years 8–14 were 86 and 85%, respectively.

**Table 2:** Demographic and military characteristics of the study population.

	**Exposed (*n* = 50,254)**	**Unexposed (*n* = 50,254)**	Adjusted RR^*^	**95% CI**
	**%**	**%**		
**Race at entry**				
White (ref)	83.54	66.95	1.00	–
Black	10.73	26.19	0.52	0.50–0.53
Other	5.73	6.86	0.81	0.78–0.85
**Education at entry**				
< HS	1.02	0.90	1.06	0.97–1.17
HS Diploma/GED (ref)	81.98	84.58	1.00	–
Some college	10.21	9.20	1.09	1.06–1.12
Bachelor's or higher	5.97	4.28	1.21	1.16–1.26
Missing	0.82	1.05	–	–
**Occupation at entry**				
Cannon crewmember	43.48	–	–	–
Combat engineer	20.38	–	–	–
Special forces	20.01	–	–	–
EOD specialist	12.89	–	–	–
Indirect fire infantry	3.24	–	–	–
Quartermaster	–	29.13	–	–
Field mechanical maintenance	–	21.40	–	–
Signal	–	13.95	–	–
Motor transport operator	–	11.05	–	–
Military intelligence	–	10.99	–	–
Military police	–	7.32	–	–
CBRN specialist	–	2.24	–	–
Engineers other than combat	–	1.97	–	–
FA radar operator/surveyor	–	1.01	–	–
Dog handler	–	0.56	–	–
Psychological operations	–	0.40	–	–
**Deployment count**				
0	41.91	41.91	–	–
1	33.02	35.46	–	–
2	15.75	14.59	–	–
3+	9.32	8.04	–	–
**Time in years to first deployment** (mean ± SD)	1.75 ± 1.21	1.90 ± 1.42	0.95	0.94–0.95
**Total months deployed** (mean ± SD)	15.23 ± 10.04	14.77 ± 9.56	1.01	1.00–1.01
**Years in service** (mean ± SD)	4.54 ± 3.12	4.87 ± 3.19	0.98	0.97–0.98

*HS, high school; EOD, explosive ordinance disposal; FA, field artillery; CBRN, chemical, biological, radiological, nuclear*.

* *Adjusted models control for race and education at military entry*.

There was no difference between the two groups for the average numbers of hospitalizations and the average number of bed days for each disease or disorder in all time periods. There was no difference in the risk of hospitalization of both groups overall and for disorder subcategory across all time periods, with the exception of a hospitalization for injury or poisoning (Table 3). The Exposed group was 16–25% more likely to be hospitalized for an injury or poisoning than the Unexposed group. When adjusted for demographic characteristics, the Exposed group continued to be more likely to be hospitalized for an injury or poisoning up to the first 7 years in service. The most common reasons for an injury/poisoning-related hospitalization in both groups were ankle fractures and heat stroke, which are not typical presentations of occupational exposure to blast. TBI diagnosis is associated with a specific traumatic event rather than chronic exposure, and in these data, TBI was not among the five most common reasons for an injury/poisoning-related hospitalization, but we included it in Table 3 as a specific diagnosis. The ICD-9 codes associated with TBI were assigned more frequently to the Exposed group than to the Unexposed group, and the adjusted risk ratio increased with time. Table 4 shows the three most common ICD-9 codes associated with TBI for each group and each time period.

**Table 3:** Risk of hospitalization overall and by disease/disorder.

	**First 12 months**				**Years 1–7**				**Years 8–14**			
	**Exposed**	**Unexposed**	aRR^*^	**95% CI**	**Exposed**	**Unexposed**	aRR^*^	**95% CI**	**Exposed**	**Unexposed**	aRR^*^	**95% CI**
	**(*n* = 50,254)**	**(*n* = 50,254)**			**(*n* = 44,520)**	**(*n* = 45,937)**			**(*n* = 6,791)**	**(*n* = 7,725)**		
	**%**	**%**			**%**	**%**			**%**	**%**		
**Any disease/disorder**	3.73	3.63	1.01	0.96–1.05	9.11	9.00	1.01	0.97–1.04	4.46	4.58	1.02	0.91–1.15
**Specific disorder**												
Headache	0.05	0.09	0.77	0.53–1.13	0.23	0.25	0.96	0.80–1.17	0.34	0.35	1.00	0.66–1.51
Sleep disturbance	0.02	0.04	0.65	0.33–1.24	0.23	0.26	0.92	0.76–1.11	0.19	0.23	0.89	0.52–1.54
Tinnitus	<0.01	0	1.83	0.36–13.02	0.02	0.01	1.25	0.65–2.40	0.04	0.01	1.42	0.46–4.39
TBI	0.08	0.05	1.23	0.90–1.66	0.32	0.17	1.32	1.12–1.55	0.16	0.03	1.85	1.02–3.34
**Disease/disorder subcategory**												
Respiratory	0.78	0.95	0.89	0.81–0.99	1.13	1.20	0.99	0.90–1.08	0.68	0.76	1.02	0.76–1.36
Nervous system/sense organs	0.23	0.22	0.99	0.83–1.19	1.32	1.13	1.07	0.99–1.16	1.24	1.20	1.03	0.83–1.28
Psychiatric	1.27	1.22	1.01	0.93–1.09	4.13	4.30	0.98	0.94–1.03	2.46	2.61	0.99	0.85–1.15
Endocrine, nutritional, immunity	0.22	0.21	1.02	0.85–1.23	0.77	0.72	1.03	0.92–1.14	0.59	0.61	1.06	0.77–1.45
Circulatory	0.25	0.21	1.10	0.93–1.32	0.95	1.04	0.99	0.90–1.09	0.88	0.91	1.00	0.76–1.31
Digestive	0.61	0.69	0.93	0.83–1.04	1.95	2.13	0.95	0.89–1.02	1.28	1.26	1.03	0.83–1.27
Symptoms/Ill-defined conditions	0.55	0.62	0.94	0.84–1.06	1.66	1.80	0.97	0.90–1.04	1.19	1.09	1.10	0.89–1.38
Injury/poisonings	1.18	0.78	1.19	1.10–1.29	3.76	2.76	1.15	1.09–1.20	1.27	0.85	1.23	0.99–1.52

* *Adjusted models control for race and education at military entry*.

**Table 4:** Most common traumatic brain injury (TBI) international classification of diseases, ninth revision (ICD-9) codes at hospitalizations.

	**Exposed**	**Unexposed**	**RR**	**95% CI**
**First 12 months**	***n*** **= 41**	***n*** **= 26**		
Concussion	43.90%	38.46%	1.14	0.63–2.07
Head injury, unspecified	21.95%	23.08%	0.95	0.38–2.36
Fracture of base of skull	9.76%	11.54%	0.84	0.21–3.48
**Years 2–7**	***n*** **= 143**	***n*** **= 76**		
Concussion	59.44%	50.00%	1.19	0.91–1.55
Intracranial injury nos	17.48%	10.53%	1.66	0.79–3.50
Fracture of base of skull	10.49%	15.79%	0.66	0.33–1.35
**Years 8–14**	***n*** **= 13**	***n*** **= 3**		
Post-concussion syndrome	38.46%	0.00%	**–**	**–**
Concussion	23.08%	100.00%	0.23	0.09–0.62
Intracranial injury nos	23.08%	0.00%	**–**	**–**

*Categories are not mutually exclusive*.

**Figure 1:**
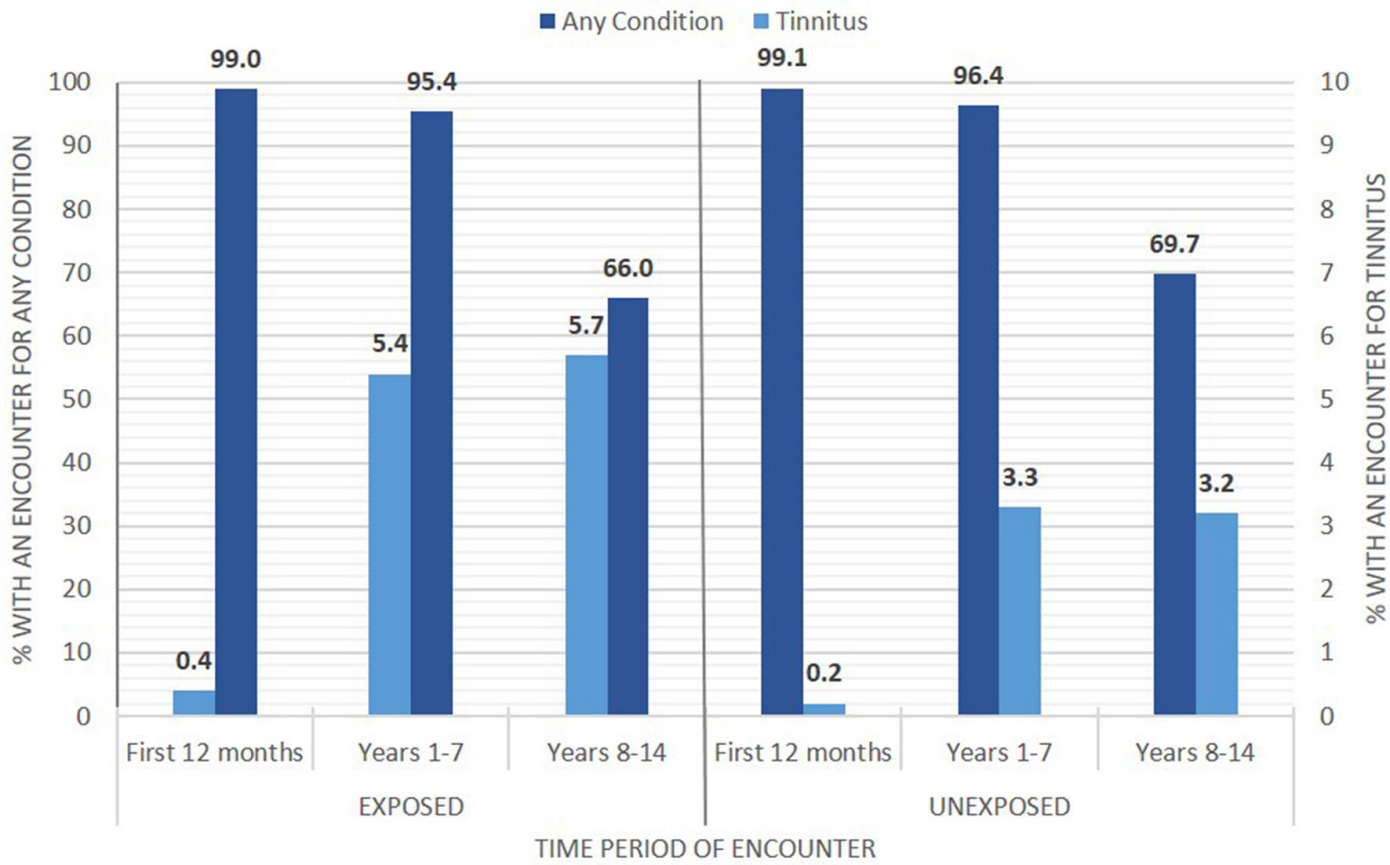
Ambulatory encounters across three time ranges of military service showing rates for tinnitus diagnosis and for any other diagnosis for each group: Exposed (left) and Unexposed (right).

Exposed Soldiers were not more likely than Unexposed Soldiers to have a larger average number of ambulatory encounters for each disorder or subcategory in all time periods. Soldiers occupationally exposed to blast had either the same or lower risk of having an ambulatory encounter both overall and for all disease or disorder subcategories than Soldiers unlikely to be exposed to blast, with the exception of diseases of the circulatory system (Table 5). The risk of an encounter for a circulatory system disease was higher in only the first 12 months of service (aRR = 1.21, 95% CI = 1.18–1.25). With regard to specific disorders likely to be associated with chronic exposure to blast, the Exposed group was more likely to have an ambulatory encounter for tinnitus in the first 12 months of service (aRR = 1.19, 95% CI = 1.03–1.37), between the first and 7th years of service (aRR = 1.21, 95% CI = 1.16–1.26) and after the 8th year of service (aRR = 1.31, 95% CI = 1.18–1.45) (Figure 1). As with the hospitalization data, TBI was not among the five most common reasons for an injury-/poisoning-related ambulatory encounter, but we included it in Table 5 as a specific diagnosis. As with the hospitalization data, the ICD-9 codes associated with TBI were assigned more frequently to the Exposed group than to the Unexposed group, and the adjusted risk ratio increased with time. Table 6 shows the three most common ICD-9 codes associated with TBI for each group and each time period.

**Table 5:** Risk of an ambulatory encounter overall and by disease/disorder.

	**First 12 months**				**Years 1–7**				**Years 8–14**			
	**Exposed**	**Unexposed**	aRR^*^	**95% CI**	**Exposed**	**Unexposed**	aRR^*^	**95% CI**	**Exposed**	**Unexposed**	aRR^*^	**95% CI**
	**(*n* = 50,254)**	**(*n* = 50,254)**			**(*n* = 44,520)**	**(*n* = 45,937)**			**(*n* = 6,791)**	**(*n* = 7,725)**		
	**%**	**%**			**%**	**%**			**%**	**%**		
**Any disease/disorder**	98.95	99.10	0.92	0.84–1.00	95.37	96.36	0.87	0.83–0.91	66.04	69.73	0.89	0.89–1.00
**Specific disorder**												
Headache	5.23	6.66	0.90	0.86–0.93	11.20	14.69	0.87	0.85–0.90	10.19	11.57	0.98	0.90–1.06
Sleep disturbance	1.23	1.53	0.88	0.81–0.95	14.42	16.84	0.91	0.89–0.94	14.86	17.02	0.95	0.89–1.02
Tinnitus	0.39	0.24	1.19	1.03–1.37	5.38	3.33	1.21	1.16–1.26	5.71	3.20	1.31	1.18–1.45
TBI	1.11	0.91	1.09	1.00–1.18	7.63	5.65	1.15	1.11–1.19	7.17	4.92	1.23	1.12–1.35
**Disease/disorder subcategory**												
Respiratory	50.34	52.40	0.96	0.94–0.97	40.04	49.51	0.82	0.81–0.84	25.36	33.79	0.82	0.77–0.87
Nervous system/sense organs	47.62	51.72	0.92	0.90–0.93	57.25	61.92	0.90	0.88–0.92	38.43	43.63	0.93	0.88–0.98
Psychiatric	18.65	20.80	0.92	0.90–0.94	49.66	52.69	0.94	0.93–0.96	35.05	37.80	0.97	0.92–1.02
Endocrine, nutritional, immunity	6.25	7.04	0.93	0.90–0.97	19.33	25.53	0.82	0.80–0.84	16.35	22.20	0.83	0.78–0.89
Circulatory	9.99	6.57	1.21	1.18–1.25	18.45	23.06	0.88	0.85–0.90	16.05	20.28	0.90	0.84–0.96
Digestive	17.23	20.93	0.89	0.87–0.91	32.00	42.55	0.79	0.78–0.81	19.76	25.22	0.87	0.82–0.93
Symptoms/Ill-defined conditions	30.65	36.20	0.88	0.88–0.91	55.81	63.56	0.86	0.85–0.88	39.36	44.88	0.92	0.88–0.97
Injury/poisonings	40.49	40.58	1.00	0.98–1.02	55.86	61.71	0.89	0.87–0.91	31.06	35.02	0.94	0.89–0.99

* *Adjusted models control for race and education at military entry*.

**Table 6:** Most common traumatic brain injury (TBI) international classification of diseases, ninth revision (ICD-9) codes at outpatient encounters.

	**Exposed**	**Unexposed**	**RR**	**95% CI**
**First 12 months**	***n*** **= 559**	***n*** **= 455**		
Concussion	53.67%	45.71%	1.17	1.03–1.33
Head injury, unspecified	37.21%	46.37%	0.80	0.69–0.93
Post-concussion syndrome	14.67%	12.75%	1.15	0.84–1.57
**Years 2–7**	***n*** **= 3,398**	***n*** **= 2,596**		
Concussion	57.83%	55.32%	1.05	0.99–1.09
Post-concussion syndrome	29.27%	26.25%	1.11	1.02–1.21
Late effect of intracranial injury w/out mention of skull fracture	19.75%	14.75%	1.34	1.19–1.50
**Years 8–14**	***n*** **= 487**	***n*** **= 380**		
Concussion	46.41%	48.16%	0.96	0.84–1.11
Intracranial injury nos	33.88%	27.89%	1.21	0.99–1.49
Late effect of intracranial injury without mention of skull fracture	33.06%	35.26%	0.94	0.78–1.13

*Categories are not mutually exclusive*.

The Exposed group was not more likely to have more prescription fills or be prescribed for a longer duration of time (days' supply) for any of the drug classes of interest than the Unexposed group at any time period in both the crude and adjusted models.

Approximately 9.5% of the Exposed group and 8.7% of the Unexposed group were evaluated for disability discharge, and the Exposed group was slightly more likely to be evaluated for disability than the Unexposed group (aRR = 1.05, 95% CI = 1.02–1.08) (Table 7). The majority of those evaluated for disability discharge in both exposure groups was separated with a one-time severance payment (48%) or medically retired (45–47%). Overall, the Exposed and Unexposed groups had a similar distribution for dispositions and ratings, and both groups had, on average, 1.7 conditions that were evaluated for disability. The group occupationally exposed to blast had a slightly shorter term of service until evaluation for disability (aRR = 0.98, 95% CI = 0.97–0.99) and had a higher risk of being evaluated for a nervous system- or sense organ-related disability between their 1st and 7th year of military service (aRR = 1.15, 95% CI = 1.07–1.25) than the Unexposed group (Table 8). No differences in the risk of disability evaluation for any of the other disease or disorder subcategories of interest in any time period met statistical criterion.

**Table 7:** Risk of disability and disability characteristics of the study population.

**Risk of disability in full population**	**Exposed (*n* = 50,254)**	**Unexposed (*n* = 50,254)**	aRR^*^	**95% CI**
	**%**	**%**		
Never evaluated for disability (ref)	90.49	91.34	1.00	–
Evaluated for disability	9.51	8.66	1.05	1.02–1.08
**Disability characteristic**	**Exposed (*****n*** **= 4,781)**	**Unexposed (*****n*** **= 4,350)**	aRR^*^	**95% CI**
	**%**	**%**		
**Disability disposition**				
Retired	47.08	45.49	1.01	0.95–1.08
Separated with severance (ref)	48.04	48.64	1.00	–
Separated w/out benefits	3.14	3.59	1.02	0.86–1.21
Fit	0.71	1.20	0.76	0.54–1.07
Other	1.02	1.06	0.99	0.75–1.32
**Combined rating**				
0	7.84	6.78	1.09	0.97–1.23
10 (ref)	26.23	26.69	1.00	–
20	13.68	15.03	0.95	0.87–1.05
30	9.12	10.32	0.95	0.85–1.06
40	7.82	6.67	1.07	0.95–1.20
50	9.16	8.67	1.00	0.90–1.12
60	6.59	6.21	1.03	0.91–1.17
70	7.74	6.87	1.04	0.92–1.17
80	2.95	3.08	0.97	0.81–1.16
90	1.23	1.01	1.06	0.81–1.38
100	2.26	2.60	0.95	0.78–1.16
Unrated	4.48	5.24	0.98	0.84–1.13
Missing	0.90	0.83	–	–
**Rating by disability eligibility**				
<30% (ref)	53.23	54.71	1.00	–
≥30% (disability retirement)	46.77	45.29	1.01	0.95–1.08
**# of conditions evaluated** (mean ± SD)	1.77 ± 1.21	1.71 ± 1.12	1.02	0.99–1.04
**Time in service to disability evaluation in years** (mean ± SD)	4.65 ± 3.09	4.86 ± 3.08	0.98	0.97–0.99

* *Adjusted models control for race and education at military entry*.

**Table 8:** Risk of evaluation for disability by disease/disorder subcategory.

**Disability disease/disorder subcategory**	**First 12 months**				**Years 1–7**				**Years 8–14**			
	**Exposed**	**Unexposed**	aRR^*^	**95% CI**	**Exposed**	**Unexposed**	aRR^*^	**95% CI**	**Exposed**	**Unexposed**	aRR^*^	**95% CI**
	**(*n* = 263)**	**(*n* = 229)**			**(*n* = 3,779)**	**(*n* = 3,401)**			**(*n* = 715)**	**(*n* = 705)**		
	**%**	**%**			**%**	**%**			**%**	**%**		
Psychiatric	6.46	3.49	1.58	0.89–2.81	26.07	25.05	1.01	0.93–1.09	55.66	48.65	1.10	0.94–1.27
Nervous system/sense Organs	7.60	7.42	1.04	0.64–1.68	20.40	15.32	1.15	1.07–1.25	29.37	23.40	1.15	0.97–1.35
Digestive	1.14	0.44	1.14	0.36–3.67	1.93	1.88	0.99	0.78–1.25	2.80	1.84	1.21	0.76–1.93
Respiratory	3.04	3.06	1.07	0.52–2.23	4.42	5.23	0.97	0.83–1.14	2.24	3.69	0.87	0.52–1.43
Circulatory	1.90	1.75	1.15	0.46–2.88	1.69	2.09	0.94	0.73–1.21	1.40	2.70	0.69	0.37–1.30
Endocrine/immunity	2.28	2.18	1.02	0.41–2.50	1.19	1.59	0.92	0.68–1.23	1.26	1.84	0.90	0.46–1.75

* *Adjusted models control for race and education at military entry*.

## Discussion

Considering the wide range and large number of endpoints considered, there were surprisingly few differences between the exposure groups that met statistical criterion. There were no substantial differences, even when statistically significant, in number of deployments, time to first deployment, duration of deployment, and time in service, and most other factors considered.

Frequency of hospitalization was rare for both groups and did not differ between exposure groups except for the injury or poisoning subcategory. Because the Exposed group included only combat arms occupations while the Unexposed group included only combat support and combat service support occupations, this difference is not unexpected. The primary reasons for injury or poisoning were ankle injury and heat stroke, which could reflect different training and operational environments. An infrequent reason for hospitalization was TBI. Although infrequent, there was a difference between groups in hospitalization for TBI in that the Exposed group showed the higher frequency at the later two time periods examined, suggesting a relationship to chronic exposure within those MOSs. TBI, in this case concussion most frequently, is associated with a specific exposure rather than chronic exposure, so the relevance of this association to the primary hypothesis is not obvious. It has been suggested elsewhere (24, 25) that chronic exposure to blast, such as is characteristic of some MOSs, may increase vulnerability to future TBI. The association in the hospitalization data we report here may be further evidence for that hypothesis.

In the ambulatory encounter data, the Exposed group had a higher frequency of circulatory system diseases; however, the risk of encounters was only higher for the first 12 months of service. For all other periods, it was lower among the Exposed group. The findings regarding circulatory diseases may reflect random statistical variation across many potential endpoints.

The findings regarding tinnitus are more interesting, as there is clear biological plausibility for a causal relationship between exposure and endpoint. The risk was higher among exposed Soldiers at every period of follow-up. Further investigation of tinnitus (see Supplemental Tables and Figure) was conducted to assess the overall risk of tinnitus diagnosis, regardless of the diagnosis order (hospitalization or ambulatory encounter). This supplemental analysis similarly found that exposed Soldiers are at an increased risk of being diagnosed with tinnitus during service [relative risk (RR), 1.75; 95% CI, 1.65–1.85], and an analysis of exposure time found the highest period of risk of diagnosis at 3–4 years of service (RR, 1.89; 95% CI, 1.62–2.18). Among those Soldiers diagnosed with tinnitus, the Exposed group was more likely to be disability discharged (RR, 1.59; 95% CI, 1.45–1.76) or attrit from service (RR, 2.42; 95% CI, 2.17–2.70) than the Unexposed group.

The findings regarding TBI are an echo of the hospitalization findings, in that there is an elevation of risk that occurs with more years of service and, assumedly, more years of exposure in the MOSs selected for occupational blast. There is an interesting difference in the ambulatory encounter data on TBI. Concussion is the most frequently appearing code, but the code for post-concussion syndrome also appears in the top three occurring codes for the majority of Soldiers with TBI. This seems reasonable because post-concussion syndrome is unlikely to result in hospitalization, but this may also be the evidence of a medical outcome associated with chronic exposure to blast. Post-concussion syndrome is associated with a specific traumatic event rather than chronic exposure, but post-concussion syndrome is divorced in time from the traumatic event, with symptoms that can be present weeks or months after injury. Furthermore, those symptoms are consistent with symptoms reported by Soldiers exposed to occupational blast (e.g., headache, dizziness, sleep difficulty, concentration difficulty). Greater frequency of post-concussive syndrome was also observed in the hospitalization data in the Exposed group for the longest time period of service, but the low number of persons in those data did not warrant standalone inference.

Exposed group members were slightly more likely to be evaluated for medical disability, but not more likely to receive any particular disability disposition or to have a higher-rated disability. They were more likely to be evaluated for a nervous system/sense organs system condition between 1 and 7 years of service, which is consistent with the observations for tinnitus in the ambulatory encounter data.

The non-specific and generally negative findings of these analyses do not support broad detrimental effects of occupational exposure to blast on health care or disability outcomes of Soldiers. The finding of tinnitus, however, does reflect specific detrimental effects that may be associated with blast exposure, particularly in the observation that odds of tinnitus diagnosis increase with apparent duration of exposure. This finding in clinical records is consistent with previous research showing self-reported tinnitus symptomology association with chronic exposure to blast (3).

The findings of TBI and post-concussion syndrome as associated with chronic blast exposure echo the pattern of tinnitus, but TBI and post-concussion syndrome are rarer conditions in these data. These findings were overlooked in initial analyses, partially due to the comparatively larger associations with musculoskeletal injury and partially due to the nature of these diagnoses' association with exposure to specific traumatic events rather than chronic exposure. In follow-up analyses, the association between exposed MOSs and TBI and post-concussion syndrome emerged. Interpreting these conditions as risks from chronic exposure to occupational blast must be considered alongside the potential that these conditions are confounding factors, potentially serving as additional causes of other conditions associated with blast exposure. That consideration seems more relevant for TBI than post-concussion syndrome, which seems more likely to be greater in frequency for blast-exposed MOS as a result of their chronic exposure. Taken together, these findings suggest particular attention to tinnitus, TBI, and post-concussion syndrome by medical personnel in evaluations of Soldiers with some routine exposure to explosives and heavy weapons, in both combat and training environments. This suggestion may not seem surprising, but this study was conducted because it was unknown if or how effects from repeated exposure to low-level blast may be captured in standard of care medical records.

Another consideration from these results, based on analyses of data drawn from standard of care medicine, is that blast-related effects from lower-level exposures may be below threshold in standard of care. The anecdotal reports and research evidence suggest that effects from low-level blast, if any, are small and accrue over long duration of repeated exposures and, thus, may require longitudinal evidence during clinical encounters for detection, especially with populations who are high functioning at baseline (26). There are current initiatives for such longitudinal assessment programs, and research evidence provides rationale for further exploration. According to Public Law 116-92 Section 717, the Department of Defense is to develop and employ a systematic means of objectively recording blast exposures among all service members or among those in select communities, yielding an exposure-based record system that would provide significant power in the evaluation of risk from exposure to blast, for both acute and chronic effects. There are limitations in epidemiological studies, and further research is warranted. A key limitation in this design was the use of MOS as a proxy for exposure to blast at an occupational level. MOS lacks precision in this approach, but there is not a separate measure of blast exposure currently available. The support for this use of MOS is in the associated training requirements and in the sample size for this study. Our use of multiple MOSs instead of a single MOS did likely introduce further limitations in precision for blast exposure history. The detonation of high explosive in training with hand grenades or in explosive breaching yields a different blast overpressure wave than does combustion of propellant in artillery or heavy weapons. However, for the purposes of the present study, we adopted the position that the differences in exposures between types of blast events were small relative to differences between our categories of MOSs, Exposed vs. Unexposed. From the examples in Table 1, following basic training, the experiences and further training for Cannon Crewmembers and Quartermasters are quite different. The size achieved for the study group increased statistical power in the analysis, needed to compare these groups for which there was no known clinical difference. The detection of a difference in tinnitus shown in these results suggests that this method for discovery was effective. In addition, this finding was consistent with a previous epidemiological study that showed association between combat arms MOSs (infantry) and risk for auditory injury (17), although that study approach used injury codes to search for MOS association rather than the approach presented here, using MOS categories to evaluate differences in injury rates. To further assess the potential for risk from occupational exposure to blast, following studies could explore annual hearing assessment results in conjunction with MOS as an additional proxy for longitudinal exposure to blast. Assays of periodically collected biological samples in the Department of Defense Serum Repository could play a similar role. Although physiological markers of low-level blast exposure have been elusive (7), there is emerging evidence that blood-based assays among personnel with a history of exposure to blast shows epigenetic differences from comparable personnel who do not have history of blast exposure (27). At minimum, the exploratory research design employed here with Soldiers could be replicated with comparable US Marine Corps populations.

In addition, the role(s) of TBI diagnoses should be followed up with studies designed for that examination. In the data presented here, TBI diagnosis occurred at rates of 7% and less across the exposure groups and time periods compared, and those comparison conditions were not balanced in size. Drawing inferences regarding TBI as an exposure effect, or cause of other outcomes, or both would be better served by a study designed for that purpose. Such future studies could also explore the role of exposure to high-energy blast exposure events in greater detail, including as design parameters length of deployment as well as time period and location of deployment. These considerations were outside the scope of the study presented here.

One further challenge encountered in the research presented here was the rate of attrition observed across the 14-year span of the studied records. The neurological insults hypothesized to result from exposure to occupational blast have been compared to chronic traumatic encephalopathy (CTE) diagnosis, and CTE among athletes has been identified as having an average latency of 15 years between exposure and symptomology, or 8 years after retirement from activity (28). In the data presented here, the average service duration for over 100,000 Soldiers was <5 years, which is consistent with other estimates of average length of service for enlisted personnel at 7 years (29). A longitudinal exposure monitoring program for active duty US military populations such as that described in Public Law 116–92 would face a challenge parallel to a challenge in the data presented in this study, the limited ability to longitudinally track health-related phenomena that have relatively long incubation periods. Veterans Affairs records may offer some advantages to protect study designs against the attrition rate observed here, but those records are not complete capture of population data in the same way as active duty military medical records. The present study was an exploration for evidence of occupational blast-related changes that reached clinical significance for active duty personnel.

In further studies of outcomes related to occupational blast exposure, low-level blast exposure, finding no clinically relevant occupational blast exposure-related effects would be welcome. Effects from blast exposure that are limited to transient phenomena (e.g., effect on immediate performance) and that are entirely reversible would be able to be managed differently. However, studies such as the one presented here are necessary to learn if injury is associated, especially as definitions of injury can change with time and advances in medical science. In the near term, this study points to opportunities for providers to monitor closely hearing conservation programs and for developers to enhance hearing protection and mitigate the elevated risk for tinnitus in these occupational blast-exposed populations.

## Data Availability Statement

The datasets generated for this study will not be made publicly available because they are drawn from medical records systems and the Privacy Act applies. Requests to access the datasets should be directed to each of the repositories identified in the article as a data source.

## Ethics Statement

The studies involving human participants were reviewed and approved by Walter Reed Army Institute of Research Institutional Review Board. Written informed consent for participation was not required for this study in accordance with the national legislation and the institutional requirements.

## Author Contributions

WC conceived of the presented idea. WC, AK, and NW developed the theory. AK, CT, and NW and performed the computations and verified the analytical methods. All authors discussed the results and contributed to the final manuscript.

## Conflict of Interest

This material has been reviewed by the Walter Reed Army Institute of Research. There is no objection to its presentation and/or publication. The opinions or assertions contained herein are the private views of the authors and are not to be construed as official or as reflecting true views of the Department of the Army or the Department of Defense. NW is an employee of the US Government. This work was prepared as part of her official duties. Title 17 U.S.C. § 105 provides that “Copyright protection under this title is not available for any work of the United States Government.” Title 17 U.S.C. § 101 defines a US Government work as a work prepared by an employee of the US Government as part of that person's official duties. The investigators have adhered to the policies for protection of human subjects as prescribed in AR 70–25. The remaining authors declare that the research was conducted in the absence of any commercial or financial relationships that could be construed as a potential conflict of interest.
