# Supplementary material for: Association of MOS-Based Blast Exposure With Medical Outcomes
Source: Front Neurol. 2020 Jul 31;11:619. doi: 10.3389/fneur.2020.00619 (PMC7413071; doi:10.3389/fneur.2020.00619)
Supplement: Supplementary file 1 [file Table_1.docx]

**Supplemental Table 1: Analysis of Exposure to Combat and Occupation in Tinnitus vs No Tinnitus, Cox Proportional Hazards**

|  | **Tinnitus (n= 4,893)** | | **No Tinnitus (n= 95,615)** | | **cRR** | **95% CI** |
| --- | --- | --- | --- | --- | --- | --- |
|  | **N** | **%** | **N** | **%** |  |  |
| **Exposure to Blast** |  |  |  |  |  |  |
| Exposed** | 2,987 | 61.0 | 47,267 | 49.4 | 1.75 | (1.65 - 1.85) |
| Unexposed (ref) | 1,906 | 39.0 | 48,348 | 50.6 | 1.00 | - |
| **MOS** |  |  |  |  |  |  |
| Cannon crewmember** | 1,476 | 30.2 | 20,372 | 21.3 | 1.65 | (1.56 - 1.76) |
| Combat engineer** | 488 | 10.0 | 9,756 | 10.2 | 1.13 | (1.02 - 1.24) |
| EOD specialist** | 278 | 5.7 | 6,199 | 6.5 | 1.09 | (0.97 - 1.24) |
| Indirect fire infantry** | 125 | 2.6 | 1,503 | 1.6 | 1.26 | (1.05 - 1.50) |
| Special Forces** | 620 | 12.7 | 9,437 | 9.9 | 1.25 | (1.15 - 1.36) |
| FA radar operator/surveyor | 21 | 0.4 | 489 | 0.5 | 0.84 | (0.55 - 1.30) |
| CBRN specialist | 46 | 0.9 | 1,080 | 1.1 | 0.86 | (0.64 - 1.15) |
| Dog handler | 6 | 0.1 | 276 | 0.3 | 0.50 | (0.23 - 1.12) |
| Motor transport operator | 311 | 6.4 | 5,241 | 5.5 | 1.06 | (0.94 - 1.18) |
| Engineers other than combat | 39 | 0.8 | 951 | 1.0 | 1.42 | (1.03 - 1.94) |
| Military intelligence | 145 | 3.0 | 5,379 | 5.6 | 0.54 | (0.45 - 0.63) |
| Military police | 213 | 4.4 | 3,464 | 3.6 | 1.25 | (1.09 - 1.44) |
| Psychological operations | 12 | 0.2 | 191 | 0.2 | 0.85 | (0.48 - 1.50) |
| Quartermaster | 499 | 10.2 | 14,139 | 14.8 | 0.59 | (0.54 - 0.65) |
| Field mechanical maintenance | 393 | 8.0 | 10,350 | 10.8 | 0.63 | (0.57 - 0.70) |
| Signal | 221 | 4.5 | 6,788 | 7.1 | 0.66 | (0.57 - 0.75) |

** Exposed Group

**Supplemental Figure 1: Time in Service of Tinnitus Cases vs No Tinnitus**

**SUPPLEMENTAL FIGURE 1 |** The highest period of risk of diagnosis for tinnitus is at 3-4 years of service and risk was higher among exposed Soldiers at every subsequent period of follow-up.

| **Supplemental Table 2: Disability Evaluation Related to Neurological or Sense Organ Condition by VASRD** | | | |
| --- | --- | --- | --- |
|  | **Exposed** | **Unexposed** |  |
| **First 12 months** | **n=20** | **n=17** |  |
| Migraine | 15.00 | 17.65 |  |
| Long thoracic nerve, paralysis | 10.00 | 0.00 |  |
| Sciatic nerve, paralysis | 10.00 | 0.00 |  |
| Hearing loss | 5.00 | 0.00 |  |
| Residuals of TBI | 5.00 | 5.88 |  |
| **Years 2 to 7** | **n=771** | **n=521** |  |
| Residuals of TBI | 23.74 | 22.46 |  |
| Migraine | 18.16 | 16.12 |  |
| Sciatic nerve, paralysis | 11.54 | 14.59 |  |
| Hearing loss | 9.08 | 4.41 |  |
| Neuritis, sciatic nerve | 3.89 | 4.22 |  |
| **Years 8 to 14** | **n=210** | **n=165** |  |
| Residuals of TBI | 36.19 | 26.06 |  |
| Migraine | 28.10 | 24.85 |  |
| Sciatic nerve, paralysis | 16.19 | 18.79 |  |
| Neuritis, sciatic nerve | 5.71 | 4.85 |  |
| Epilepsy, grand mal | 4.76 | 4.85 |  |
|  |  |  |  |
| Listed the top 5 VASRDs for Exposed group, then found the % for the same conditions in Unexposed (not necessarily top 5 VASRDs for Unexposed group). | | | |
|  | |  |  |
